# Material, Aerodynamic, and Operational Aspects of Single-Skin Paraglider

**DOI:** 10.3390/ma17225553

**Published:** 2024-11-14

**Authors:** Paulina Maślanka, Ryszard Korycki

**Affiliations:** Department of Mechanical Engineering, Informatics and Chemistry of Polymer Materials, Lodz University of Technology, Żeromskiego 116, 90-924 Lodz, Poland; ryszard.korycki@p.lodz.pl

**Keywords:** single-skin paraglider, packing volume, aerodynamics, structural analysis, numerical modeling

## Abstract

The operating comfort of a paraglider is created by the aerodynamic parameters as well as the mass and packing volume of the wing. A classic paraglider has upper and lower covers. To reduce the material and manufacturing costs as well as protect the environment, it is possible to introduce a single-skin wing. This article conducts an analysis of a single-skin paraglider covered only with upper panels, whereas the lower cover is applied only at the leading and trailing edges. The analysis is theoretically oriented; aerodynamic and structural calculations were performed using the ANSYS environment. The single-skin structure was evaluated in terms of the predicted behavior during flight and the material’s deformation under the influence of a specified pressure and the overloads acting on it. The results show that developing these structures may influence the creation of models with comparable aerodynamic characteristics to traditional ones. Additionally, the reduced masses and packing volumes of difficult-to-degrade materials are strongly correlated with saving costs and an ecological approach. No corresponding studies were found in the available literature. Thus, this presented analysis may result in a greater understanding and application of this paraglider type.

## 1. Introduction

Today, manufacturers of sports equipment are aiming to design products with minimized masses and volumes; one of the key roles here is assigned to materials engineering. There are many objectives of such actions, e.g., the maximization of the achievements of the athlete, reductions in costs and materials used, which translates into a better ecological approach, etc.

In paragliding, the most common wing geometry is a traditional type, with full textile covering (lower and upper). However, a few single-skin models (with an upper cover only) can be found that meet the objectives described above [1,2]. Despite its availability on the market, this type of wing has not been found to be studied in any studies in the scientific literature.

**Figure 1:**
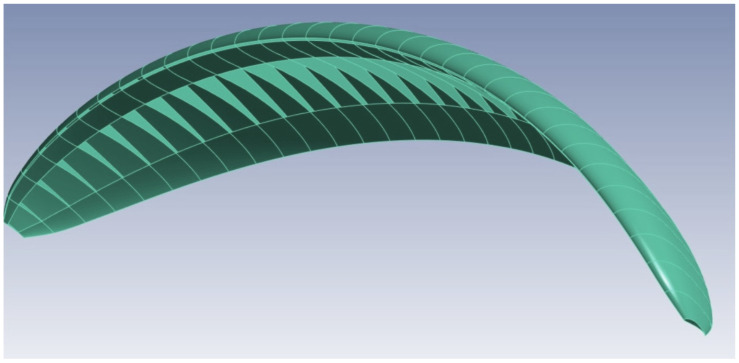
The geometry of the considered wing structure.

This article conducts an analysis of a paraglider covered only with upper panels, as shown in Figure 1. The lower cover is applied at the leading and trailing edges, which initiates the airstream path as well as maintains the shape of the airfoil.

This study is focused on the effects of the type of material and geometry on the mass and packing volume of the final product; it also conducts an evaluation of the proposed solution in terms of the paraglider’s behavior in flight and the material’s deformation under the influence of pressure and the overloads acting on it.

Both aerodynamic and structural calculations were performed using the ANSYS software 2022R2 release (i.e., the ANSYS Fluent and ANSYS Structural programs). The use of this program enabled us to [3,4,5] (1) visualize the results, (2) obtain clear data regarding the variables correlated with the model’s behavior, and (3) save costs related to creating real conditions.

The available scientific literature analyzing paraglider wings includes experimental [6,7,8,9], numerical [10,11,12,13], and general (connecting experimental and numerical approaches) [14,15] studies. 

None of the above-listed experimental methods were found to be necessary to implement in this study as it is an introduction to the considerations of single-skin paragliders. However, wind tunnel testing [8,9], as well as an assessment of the paraglider’s shape and deformation in flight [7], will be important approaches when covering this subject in the future. Moreover, as shown in [14,15], combining experimental and numerical methods is a common approach, which supports the validation of the results and helps in applying the most appropriate mesh, turbulence model, etc.

Computational fluid dynamics (CFD) may not only help in the analysis of the wing overall [13] but also in the analysis of the characteristic elements of the canopy, such as the air inlet [12,16], arc-anhedral angle [11], and airfoil [10,11,14,16]. The geometry proposed in the present study is an initial and non-optimized version; any further studies on the model will require both general and detailed analyses of all elements of the paraglider system.

An approach taking into consideration the covering material’s properties and its influence on the paraglider’s behavior has only been found in the authors’ previous publications [17,18,19].

The literature review revealed that previous studies regarding paragliders are not applicable to this article. However, the development of the topic of single-skin paragliders in the future may require using the above-listed methods and their modifications.

As mentioned above, an analysis of a single-skin wing has significant importance in view of the packing volume and mass of the final product. After applying the new geometry, in each case, the masses and packing volumes would decrease by around 26% compared to the previously considered paraglider. 

The novelty elements of this study are the following: (I) it presents a new cost- and material-saving paraglider wing type in terms of the scientific approach; (II) it displays the potential of developing single-skin models while maintaining aerodynamic performance; and (III) it employs an interdisciplinary approach involving the synergy of materials engineering, environmental protection, the mechanics of materials, and the aerodynamics of a new paraglider structure.

Moreover, this problem can be expanded by many approaches. One interesting direction for future studies regarding this type of wing may be the implementation of geometry in studies in turbulent atmospheres [20] in order to predict the paraglider’s behavior more precisely. A way to improve the aerodynamic characteristics of the proposed solution may be by performing the multi-criteria optimization of, e.g., geometry elements (e.g., the airfoil shape, the surface of the wing, and inlet placement). This can be achieved by using data aggregation techniques, such as multi-dimensional extensions of the Choquet integral, as manifested in [21]. Another direction may be to improve the properties of the covering material [22].

## 2. Materials and Methods

### 2.1. Materials

According to [18,19], ten Polyamide 6.6 woven rip-stop fabric samples, differing mainly in mass and thickness, were used for comparison purposes regarding the mass and packing volume of the final product. Sample no. 2 was selected for further aerodynamical and structural analyses. 

The general properties of the fabrics are presented in Table 1. More insightful studies of the same materials were described in the previous literature, which acts as an introduction to this research [18,19].

As shown in Table 1, the analyzed surface mass of the samples ranged between 26 g/m^2^–and 42 g/m^2^, and the thickness ranged between 0.04 mm–and 0.09 mm. The increased masses and thicknesses of the samples were usually associated with the increase in maximum force during elongation.

As mentioned above, sample no. 2 was selected for the structural calculations. Therefore, based on the data listed in Table 1, the critical values of stress, strain, and deformation were calculated and listed in Table 2 below.

### 2.2. Methods

#### 2.2.1. Determination of Expected Masses and Packing Volumes of the Wing

In order to determine the expected masses and packing volumes of the considered paraglider wing, the following data were used: wing surface, considering the upper and bottom surfaces and ribs (*S*); the surface mass of the applied material (*m_s_*); and the thickness of the applied material (*t*). 

The wing surface was exported from the ANSYS Design Modeler Release 2022R2 after generating the final geometry of the wing. The material properties were obtained as a result of previous studies [18,19] and can be found in Table 1.

The surface of the materials used in the production of a conventional type of paraglider wing (including brits and ribs) is equal to 71.65 m^2^, whereas, in the case of the paraglider considered in this study, it is 53.02 m^2^. This results in around a 26% decrease in final masses and packing volumes in each case of the considered material.

The masses and the packing volumes of the final product were calculated according to the below Equations (1) and (2).

(1) $$M_{x}=S\cdot m_{s}$$

(2) $$V_{x}=S\cdot m_{s}\cdot t$$

where

*M_x_*—mass of the paraglider wing [kg];
*S*—summary surface of the upper surfaces, bottom surfaces, and ribs forming the wing [m^2^];
*m_s_*—surface mass of the applied material [kg/m^2^];
*V_x_*—packing volume of the paraglider wing [m^3^];
*t*—thickness of the applied material [m].

#### 2.2.2. Computational Analysis

As mentioned above, the calculations were performed using the Fluent and Structural programs in the ANSYS software (2023R1 release). Due to the application of two numerical methods, the creation of the calculation geometry domains, as well as meshes, differed in the considered case. 

For both, geometries were generated using the ANSYS Design Modeler Release 2023R1.

For the ribs’ shape definition, the under-cambered type of Selig S1223 airfoil [23] was selected. The previous studies of the authors [17,18,19] used the conventional airfoil types in order to establish the geometry. The airfoil type change in the present analysis was caused by the significantly different nature of the newly studied paraglider. The rib shape of traditionally used airfoils (asymmetrical or flat bottom) would result in creating additional drag related to the open wing bottom in comparison to the under-cambered one. 

All the applied dimensions (span, chord, radius, etc.) characterizing the studied paraglider wing were the same as the ones in the previous papers [18,19]. Therefore, the following steps of establishing the geometry for both computational fluid dynamics (CFD) analysis and structural calculations performed by the finite elements method (FEM) also remained comparable and hence will not be widely described in this article.

The selected software for CFD analysis involves the use of the finite volume method, assuming that if the mass, energy, and momentum balance for each elementary cell is satisfied, then the balance in the entire calculation volume is also met. Therefore, nonlinear differential equations describing the conservation of mass, energy, and momentum are numerically solved.

Unlike the previous studies [17,18,19], in the present case, the ribs were not only created as a basis for the 3D geometry but also considered in the numerical calculations regarding the flow. 

The presence of ribs in the implemented geometry is vital, as the surfaces at the bottom of the designed wing were not closed. In comparison, in the earlier studies, the airfoil shape was maintained by the lower and upper surfaces. Thus, in the case under consideration, a modeled airfoil shape had a significant influence on the final results of the CFD calculations. When the geometry was generated, each segment of the paraglider created a separate body. 

The generated geometry of the multibody part contained surfaces creating the paraglider wing and body amounting to the calculation volume. The dimensions of the cuboid surrounding the paraglider remained the same, as in the above-mentioned articles [15,16], thus 20 m in front of the wing and 40 m in other directions.

**Figure 2:**
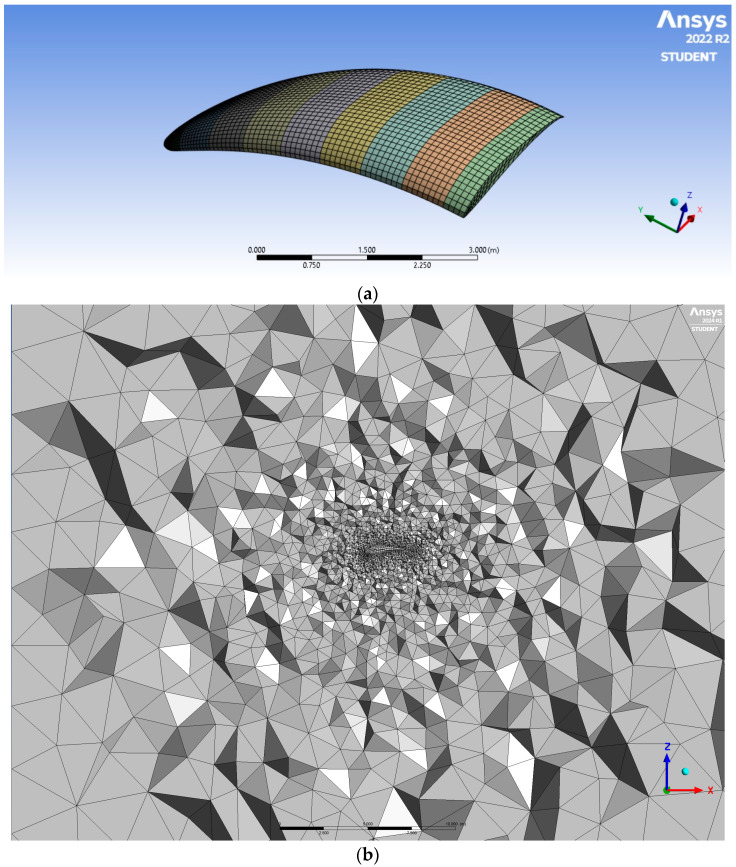
The generated mesh: (**a**) structural elements forming the paraglider wing and the air inside; (**b**) tetrahedral elements in the area surrounding the object.

The geometry was brought into ANSYS Meshing Release 2022R2 in order to generate a mesh dedicated to the finite volume method. The hybrid mesh was prepared. It was built mostly with hexahedral elements forming the paraglider wing, the air inside (Figure 2a), as well as tetrahedral elements in the area surrounding the object (Figure 2b); pyramidal elements were implemented between the two zones.

The generated mesh was created from 350,556 elements. Their average orthogonal quality was equal to 0.75 at minimum value at the level of 0.1. The average skewness metric was equal to 0.25, whereas its maximum value was 0.90. The minimum and maximum element volumes were equal to, correspondingly, 9.178503 × 10^−9^ m^3^ and 7.892048 m^3^.

The types of used boundary conditions are listed in Table 3. The description of each boundary condition can be found in previous articles [14,15].

The pre-processing in the ANSYS Fluent program was performed according to the steps described previously [14,15]. Initially, the angle of attack remained at the same level, i.e., α = 6°. However, it was found that for this type of geometry, the most advantageous angle of attack was equal to 12°, as it allowed to obtain an increased c_l_/c_d_ ratio. Thus, the further calculations considered the following conditions: pressure 101,325 Pa; temperature: 26.85 °C; viscosity: 1.7894 × 10^−5^ kg/(m·s); density: 1.177 kg/m^3^; velocity: 45 km/h; angle of attack: 12°. 

The selected medium was air as an ideal gas. Considering the type of analysis, the viscosity of air was set to be constant. The walls limiting the paraglider geometry were nonporous and of infinitesimal thickness (no material assignment was required). 

The pressure-based solver was utilized. The Spalart–Allmaras turbulence model and the third-order-MUSCL discretization scheme were applied. Implementing such settings allowed for a better comparison between the new and the traditional types of paraglider [18,19]. Moreover, due to its characterization, the Spalart–Allmaras turbulence model was found to be possibly the most advantageous for these types of flying objects and the implemented mesh [18].

To perform the structural calculations, it was enough to present a shell-type geometry of the wing, omitting the calculation volume. The ribs constituted the fixed supports, whereas the remaining surfaces were the subject of structural analysis. The load applied to act on the surfaces and covering materials was determined by the pressure distribution over the paraglider wing (obtained in the Fluent program), as well as an overload assumed to be equal to 5.6 [18,19].

The generated mesh was formed from 31,514 elements, with the following metrics: element quality = 0.95; skewness = 0.06; aspect ratio = 1.08. The mesh was mainly structural, and only some of the mesh elements remained marginally skewed.

The result was obtained by implementing the Newton–Raphson method. This approach is used when dealing with nonlinear force displacements. At the start, the system is stationary. Next, consecutive iterations of displacements and forces were performed until the convergence was reached.

The Newton–Raphson procedure can be described by the following formulas [5] concerning the displacement increment and residual force:

(3) $$\Delta u^{n}=(F_{ext}-F_{int}^{n})/K^{n}$$

(4) $$F_{res}^{n}=F_{ext}-F_{int}^{n}$$

where

Δ*u*—displacement increment [m];
*n*—iteration (subsequent) [-];
*F_ext_*—applied force [N];
*F_int_*—computed internal force [N];
*F_res_*—residual force [N];
*K*—stiffness matrix [Pa].

Only fabric no. 2 was selected for the structural analysis. The reason for this choice is that this fabric was the strongest among all the materials considered. However, an increased angle of attack to be applied for this type of geometry was expected to create a greater load acting on the paraglider cover. Based on the material properties listed in Section 2.1., the new material was designed and added to the engineering data of the Ansys Structural Project. The remaining properties were established on the basis of the available literature [24]. 

## 3. Results

### 3.1. Masses and Packing Volumes Comparison

Masses and volumes of a paraglider wing with respect to the materials used were evaluated and are listed in Table 4 (based on the methods described in Section 2.2.1).

The determined lowest masses of the single-cover wing and the traditional wing were, respectively, equal to 1.378 kg and 1.863 kg; those results were obtained from the lightest materials (no. 4 or no. 8). Therefore, the minimum mass decrease caused by the change of geometry was equal to 0.485 kg. 

When the packing volumes were analyzed, the lowest values were obtained for samples no. 7 and no. 8: 2.121 dm^3^ (single-cover paraglider) and 2.866 dm^3^ (traditional paraglider). The decrease in volume caused by the geometry change was equal to 0.745 dm^3^ when using one of the fabrics.

It is worth noting that the maximum savings can amount to 0.782 kg in mass (fabrics no. 2, 10) and 1.677 dm^3^ in packing volume (fabrics no. 5, 6).

However, the discussion above considered only the working surfaces and did not include additional elements of the wing, in particular, fabric allowances, sewing threads, and plex stiffening elements. Therefore, in both cases (single-skin and traditional wing), the actual masses and volumes would be significantly increased compared to the presented results. 

Moreover, it should be noted that hand packing is used for paragliders, whereas the obtained packing volumes were based on the surfaces and thicknesses of the covering materials and ribs. It means that the calculated volumes would only be possible to obtain if the under-pressure packing method was used.

### 3.2. Computational Simulations

The aerodynamic calculations described in Section 2.2.2 were stopped after the convergence achieved at 850 iterations. The obtained results regarding the lift and drag forces, as well as the c_l_/c_d_ ratio, are compiled in Table 5. The lift force, drag force, and **c_l_/c_d_** ratio are aerodynamic characteristics, which are further discussed in [14].

The analysis of the values obtained for the new type of geometry demonstrates that the lift and drag forces were significantly increased in comparison to those obtained for a traditional wing. As already mentioned in Section 2.2.2., this was expected as a result of applying a greater angle of attack.

These values were decided not to be validated experimentally nor analytically at this stage of the study. The presented analysis has an introductory character, and the simulations were performed for the initial assessment of the proposed solution. Future studies will entail the implementation of experimental validation. The analytical validation with the use of general formulas would not cover such elements as wing deflection or open bottom. Thus, this validation method would present inaccurate lift and drag forces, significantly differing from those numerically obtained here. 

**Figure 3:**
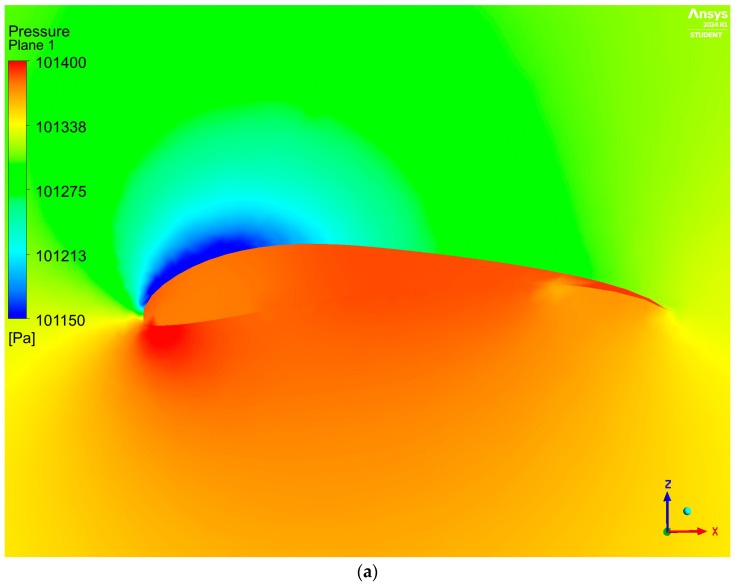

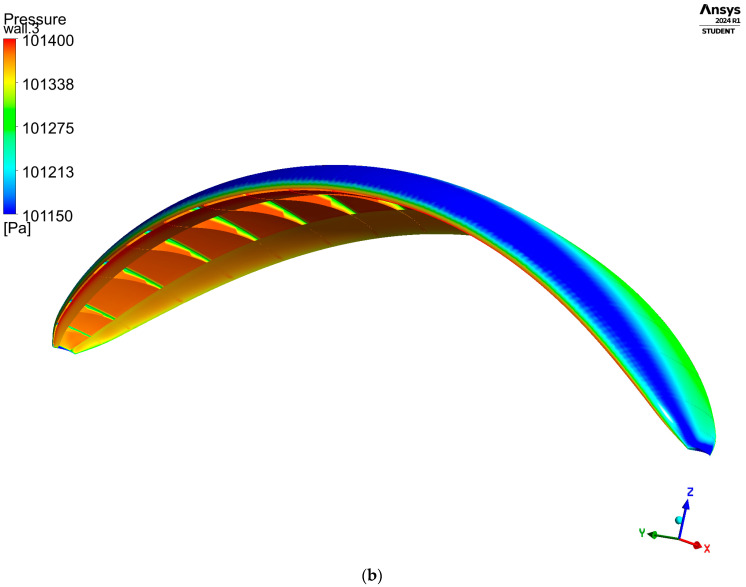
Pressure distribution over the considered geometry: (**a**) in the symmetry plane; (**b**) on the walls of the paraglider.

The pressure distribution around the considered wing is shown in Figure 3.

According to the pressure distribution presented in Figure 3, it can be concluded that the pressure inside the wing was distributed unevenly; this was never observed for the traditional type of geometry covered with an impermeable material. However, this type of pressure distribution can be explained by the type of geometry under consideration (surfaces do not form closed spaces at the bottom).

Moreover, a substantial decrease in pressure above the wing was noted. This change was another result of applying an increased angle of attack. 

The overpressure created outside the wing around its nose was greater compared to the one created on the inner surface in this region. However, it was not observed entirely from the front. Therefore, the initial observation indicates that maintaining the designed shape of the wing would be undisturbed by pushing the leading edge to the inside.

An increased pressure acting on the lower surface of the paraglider was observed; this could also cause distortion of the original geometry of the wing by pushing the covering material to the inside. However, placing ribs every 0.3 m would provide support to the bottom material at the front. Moreover, a significant distance between the upper and lower covers in this section would prevent mutual suction of these layers.

A huge overpressure at the trailing edge inside the wing ensured the maintenance of the shape in this section. The distance between the upper and the lower covers in this section was insignificant; different distributions would cause mutual suction of both layers and, therefore, distortion of the shape of the airfoil. Thus, the overpressure created there is very important for safety reasons.

The last important observation regarding pressure distribution pertains to the ribs, as they were assessed to be uneven (Figure 3b). Such behavior can be concerning. However, it is worth noting that the modeled wing presents a simplified case without holes in the ribs. In paragliders available on the market, holes are present in the ribs in order to allow the air to be distributed evenly. 

**Figure 4:**
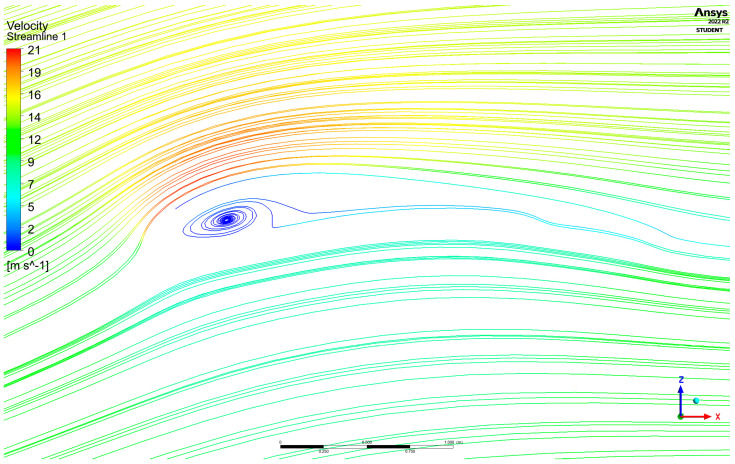
Streamlines of velocity are colored by velocity magnitude (symmetry plane).

Streamlines of velocity colored by velocity magnitude can be seen in Figure 4 below.

Airstreams of an increased velocity were observed in the vicinity of the upper surface, which is consistent with the pressure distribution and the Bernoulli equation [25]. A circular motion of airstreams formed inside the paraglider, which was not noticed in the previously analyzed traditional geometries [17,18,19]. As its speed was close to 0 m/s, the observed phenomenon was initially assessed as safe for the usage of the considered wing. The created circular motion can be explained by the different angle of attack, the airfoil type change, or the opened bottom surface. It was the only flow disturbance that was observed at this level of analysis.

Nevertheless, as paragliders are composed of woven fabrics, they have no rigid structure. Therefore, they are more sensitive to any disturbances (caused by gusts, vortices, or evolutions performed by a user) than other flying objects. Thus, regardless of the promising initial assessment, further studies are necessary.

Numerical calculations and measurements that would be useful in the future are the following: mesh sensitivity analysis (assuring the accuracy of the obtained results), study on the mid-flight safety in turbulent atmosphere, the introduction of the dynamic mesh (the behavior of an unrigid structure), etc. The certification process required for each paraglider model introduced on the market would additionally protect from any danger caused by the structure.

**Figure 5:**
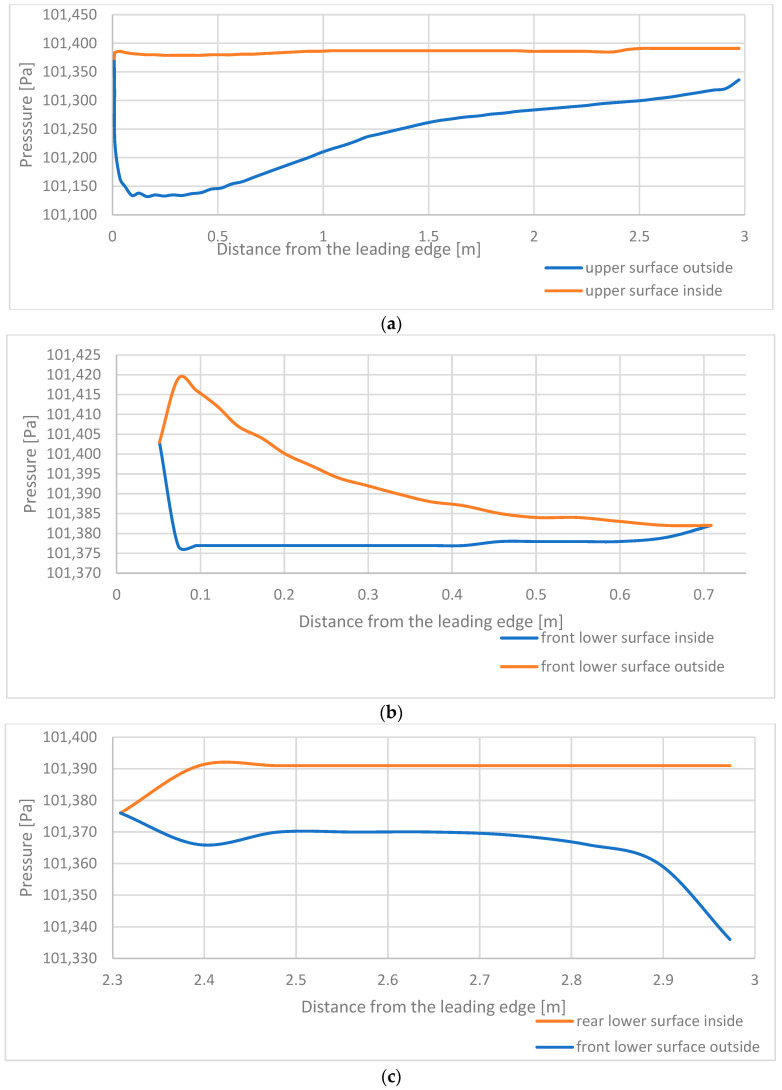
Pressure acting on a material in the function of distance from the leading edge in the symmetry plane: (**a**) upper surface; (**b**) front lower surface; (**c**) rear lower surface.

Figure 5 showcases the pressure acting on a material in the function of distance from the leading edge in the symmetry plane. The data were introduced to the structural calculations with the assumption of an overload equal to 5.6, which is a maximum temporary overload that the paraglider is expected to be exposed to, as was further explained in [18,19].

**Figure 6:**
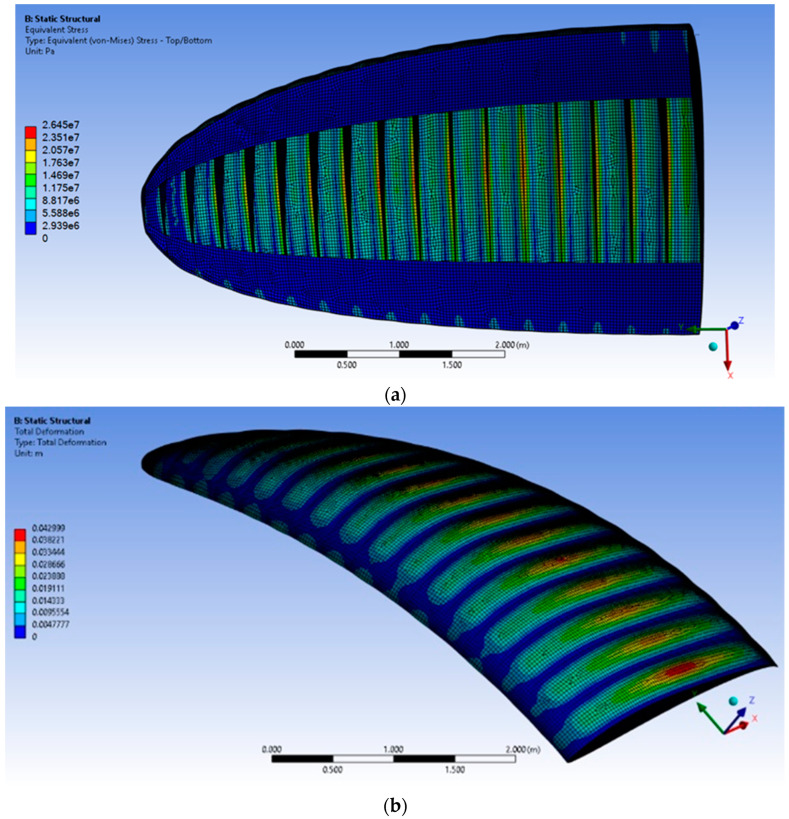
Distribution of stress and deformation over the considered type of paraglider: (**a**) stress from the bottom view; (**b**) deformation from the axonometric view.

In the course of numerical calculations, stress, strain, and deformation results of the woven fabric no. 2 were obtained. Their distribution over the wing geometry is presented in Figure 6. The numerical results are compiled in Table 6.

The behavior of the material was observed to be like that of the ones in the traditional type of paraglider, i.e., the greatest deformation was noticed in the middle distance between the ribs, and the highest cumulation of stress was found to be in the nearest regions of the ribs. 

However, a minor difference in the deformation distribution was observed, similar to the previous cases, in which greater values on the paraglider’s leading edge were noted [18,19]. Nevertheless, in the present analysis, the highest deformation level was found in the middle section of each material segment (brit). Notably, that deformation is strongly associated with pressure distribution, which also presented many differences for both the traditional and the new type of paraglider. Therefore, the deformation distribution change can be explained by the new nature of the geometry.

The mid-flight safety of a single-skin paraglider may be predicted from the deformation of the material. As expected from the CFD calculations, no dangerous deformation of the bottom parts was observed when the structural results were analyzed.

**Figure 7:**
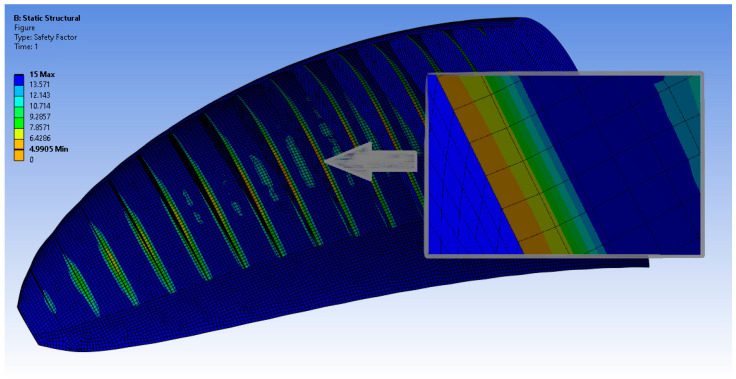
Safety factor in areas of stress concentration (lowest values of safety factor).

The results compiled in Table 6 show similar dependencies to those analyzed in [18,19]. However, for this geometry, the maximums were greater. This is associated with the implementation of an increased angle of attack and the resultant higher values of the initial pressure acting on the paraglider surfaces. The determined safety factor based on the material properties and stress maximum value was equal to 4.99 (Figure 7). For airplanes, safety factors at the level of 1.4 or greater are considered as safe. Thus, the initial assessment of the analyzed model was positive. 

The safety of the analyzed structure was also confirmed on the basis of a comparison of the critical strain and deformation values compiled in Table 2 (Section 2.1.) with the results listed in Table 6. 

## 4. Conclusions

The aim of this paper was to model an innovative paraglider single-skin wing type and analyze it with respect to the minimization of materials used for its production, as well as an assessment of the model’s general behavior mid-flight (based on the Fabric no. 2 example, i.e., its aerodynamic performance, global deformation, and stress determination).

In the first part of the analysis, masses and volumes of a paraglider wing with respect to 10 different paragliding materials used were evaluated. They were compared to the values present in a traditional type of wing. As the surfaces of the materials used in the production of a conventional type and single-skin wings (including brits and ribs) are, respectively, equal to 71.65 m^2^ and 53.02 m^2^, the mass and volume decreases for each considered material may be around 26%. In the case of the listed materials, savings achieved by applying the new geometry ranged from 0.485 kg–0.782 kg (on mass) and 0.745 dm^3^–1.677 dm^3^ (on volume). The observations showed that from the ecological and economical approach, it is very important to popularize the single-skin models. Moreover, in some types of paragliding activities (e.g., para-hiking), the decreased mass and packing volume models are preferred by the users. 

The results obtained in the course of the numerical calculations showed that the c_l_/c_d_ ratio of the proposed geometry was equal to 11.27 (angle of attack α = 12°), which is a decrease when compared to the traditional wing performance (13.17 with α = 6° [18,19]). However, the result is still satisfactory. It is worth mentioning that the considered geometry has not been optimized (it was the only geometry newly created by the authors). Therefore, it is expected that some single-skin models may reach much more increased values of the c_l_/c_d_ ratio. 

The pressure distribution and streamlines of the velocity figures were analyzed to assess the safety aspects of the flight. No alarming features were observed. 

The structural calculations were performed only for material no. 2, as it was the strongest among all the samples, and were performed with the assumption of an overload equal to 5.6. The biggest cumulation of stress in the material in the modeled case was observed in the region of implemented supports (ribs); the greatest deformation was noted in the middle of each segment (brit). The numerical results showed that the safety factor calculated for this case was a value of 4.99.

As mentioned in the introduction, some paraglider manufacturers introduced single-skin models. However, such models are still a minority, dedicated to a narrow group of users. Moreover, this type of geometry was not found to be studied in any research. 

The satisfactory results show that developing this type of structure may lead to the creation of models of comparable aerodynamic characteristics to the traditional ones; the additional benefits are reduced masses and packing volumes, which are strongly correlated with saving costs and an ecological approach. 

The methods presented in this publication can support the selection of materials that would be the safest and most advantageous for the new types of wings when both the geometry and the requirements of the final product are considered.

The problem will be further developed by implementing the model into a turbulent atmosphere and introducing a multi-criteria optimization of geometry elements in order to increase the performance and safety of the wing.

## Figures and Tables

**Table 1:** Basic characteristics of the analyzed materials.

Sample	Surface Mass [g/m^2^]	Thickness [mm]	Number of Threads/1 dm		Max Force During Elongation [daN/5 cm]		Elongation at Break [%]		Air Permeability (Pressure Drop 200 Pa)
			Warp	Weft	Warp	Weft	Warp	Weft	
1	34	0.05	560	580	34	32	21	20	0.00
2	42	0.07	460	500	47	46	30	30	0.00
3	32	0.05	420	460	25	33	24	25	0.00
4	26	0.05	420	580	25	22	24	22	0.00
5	38	0.09	420	480	38	33	25	25	0.00
6	38	0.09	420	460	27	28	21	23	0.00
7	29	0.04	420	460	25	33	24	25	0.00
8	26	0.04	420	480	25	22	24	23	0.00
9	36	0.05	520	520	40	40	26	26	0.00
10	42	0.08	510	500	42	42	27	27	0.00

**Table 2:** Critical values of stress, strain, and deformation (Sample no. 2).

Sample	Stress [Pa]		Strain [%]		Deformation [m] *	
	Warp	Weft	Warp	Weft	Warp	Weft
**Fabric no. 2**	1.343 × 10^8^	1.314 × 10^8^	30	30	0.154	0.154

* for segment width equal to 0.3 m.

**Table 3:** Types of boundary conditions.

Geometry Element	Boundary Condition
Wing upper cover	wall
Wing bottom cover (leading edge area)	wall
Wing bottom cover (trailing edge area)	wall
Wing ribs	wall
Cross-sectional surface	symmetry
External surfaces of the calculation domain	pressure far field

**Table 4:** Expected masses and volumes of a wing comparison with respect to the applied geometry and covering materials.

Sample No.	Mass (Traditional Type of Paraglider) [18,19]	Mass (New Type of Paraglider)	Mass Decrease	Packing Volume (Traditional Type of Paraglider) [18,19]	Packing Volume (New Type of Paraglider)	Packing Volume Decrease
	[kg]	[kg]	[kg]	[dm^3^]	[dm^3^]	[dm^3^]
1	2.436	1.802	0.634	3.582	2.651	0.931
2	3.009	2.227	0.782	5.015	3.711	1.304
3	2.293	1.696	0.597	3.582	2.651	0.931
4	1.863	1.378	0.485	3.582	2.651	0.931
5	2.723	2.014	0.709	6.448	4.771	1.677
6	2.723	2.014	0.709	6.448	4.771	1.677
7	2.078	1.537	0.541	2.866	2.121	0.745
8	1.863	1.378	0.485	2.866	2.121	0.745
9	2.579	1.909	0.670	3.582	2.651	0.931
10	3.009	2.227	0.782	5.732	4.241	1.491

**Table 5:** Obtained results regarding CFD calculations of single-cover and traditional wings.

	Single-Cover Wing	Traditional Wing [18,19]
	v = 45 km/h; *p* = 101,325 Pa; α = 12°	v = 45 km/h; *p* = 101,325 Pa; α = 6°
Lift force	3472 N	1519 N
Drag force	307 N	115 N
c_l_/c_d_	11.27	13.17

Where according to [14,15]: v—flying speed [km/h], *p*—surface stress/pressure [Pa], and α—angle of attack [°].

**Table 6:** Obtained results regarding CFD calculations of single-cover and traditional wings.

Sample	Stress [Pa]			Strain [%]			Deformation [m]		
	Min.	Av.	Max.	Min.	Av.	Max.	Min.	Av.	Max.
Fabric no. 2 Single-cover wing	0.000	3.272 × 10^6^	2.645 × 10^7^	0.0	0.6	6.8	0.000	0.006	0.043
Fabric no. 2 Traditional wing	0.000	3.598 × 10^6^	2.220 × 10^7^	0.0	0.9	6.4	0.000	0.008	0.033

## Data Availability

The original contributions presented in this study are included in the article. Further inquiries can be directed to the corresponding authors.
